# The genome sequence of the dun-bar pinion, *Cosmia trapezina* (Linnaeus, 1758)

**DOI:** 10.12688/wellcomeopenres.17925.1

**Published:** 2022-07-13

**Authors:** Douglas Boyes, University of Oxford and Wytham Woods Genome Acquisition Lab, Darwin Tree of Life Barcoding collective, Wellcome Sanger Institute Tree of Life programme, Wellcome Sanger Institute Scientific Operations: DNA Pipelines collective, Tree of Life Core Informatics collective, Charles Godfray, Peter W. H. Holland, Darwin Tree of Life Consortium

**Affiliations:** 1 UK Centre for Ecology and Hydrology, Wallingford, Oxfordshire, UK; 2 Department of Zoology, University of Oxford, Oxford, Oxfordshire, OX1 3SZ, UK

**Keywords:** Cosmia trapezina, dun-bar pinion, genome sequence, chromosomal, Arthropoda

## Abstract

We present a genome assembly from an individual male *Cosmia trapezina* (dun-bar pinion; Arthropoda; Insecta; Lepidoptera; Noctuidae). The genome sequence is 825 megabases in span. The majority of the assembly (99.87%) is scaffolded into 32 chromosomal pseudomolecules with the Z chromosome assembled. The complete mitochondrial genome was also assembled and is 15.4 kilobases in length.

## Species taxonomy

Eukaryota; Metazoa; Ecdysozoa; Arthropoda; Hexapoda; Insecta; Pterygota; Neoptera; Endopterygota; Lepidoptera; Glossata; Ditrysia; Noctuoidea; Noctuidae; Ipimorphinae; Cosmia; Cosmia trapezina (Linneaus, 1758) (NCBI:txid116126).

## Background

*Cosmia trapezina,* the dun-bar, is a medium-sized moth in the large family Noctuidae. It is found throughout most of the Palaearctic and in Britain is considered a common species. The forewings of *C. trapezina* are variable in colour, though the broad sharply-angled band running laterally, the ‘dun bar’, is characteristic, and this is an easy species to recognise.

To look at, the dun-bar is an unremarkable moth, but it has an unsavoury reputation. Lepidopterists learning to collect and rear caterpillars are taught to recognise dun-bar larvae and segregate them from other species and ideally rear them individually. Bucking the vegetarianism of most moths, the dun-bar has a carnivorous bent and while it will survive and grow on a diet of tree leaves it will eat the larvae of other moth species, notably *Operophtera brumata* (the Winter Moth), and will also cannibalise its own species. Newman in 1869 described *C. trapezina* larvae chasing prey and seizing them behind the head “with savage eagerness” ( Newman, 1869). A few other species share this tendency, notably its North American relative *C. calami* ( Donald Lafontaine & Troubridge, 2003) and there is a clade of Hawaiian pug moths, *Eupithecia* sp., that are obligate predators ( Montgomery, 1983). Eating your competitors has been termed intraguild predation ( Polis, 1981) and there is speculation that the dun-bar’s proclivities may affect insect community structure in European woodlands ( Turčáni & Patočka, 2011).

## Genome sequence report

**Figure 1.**
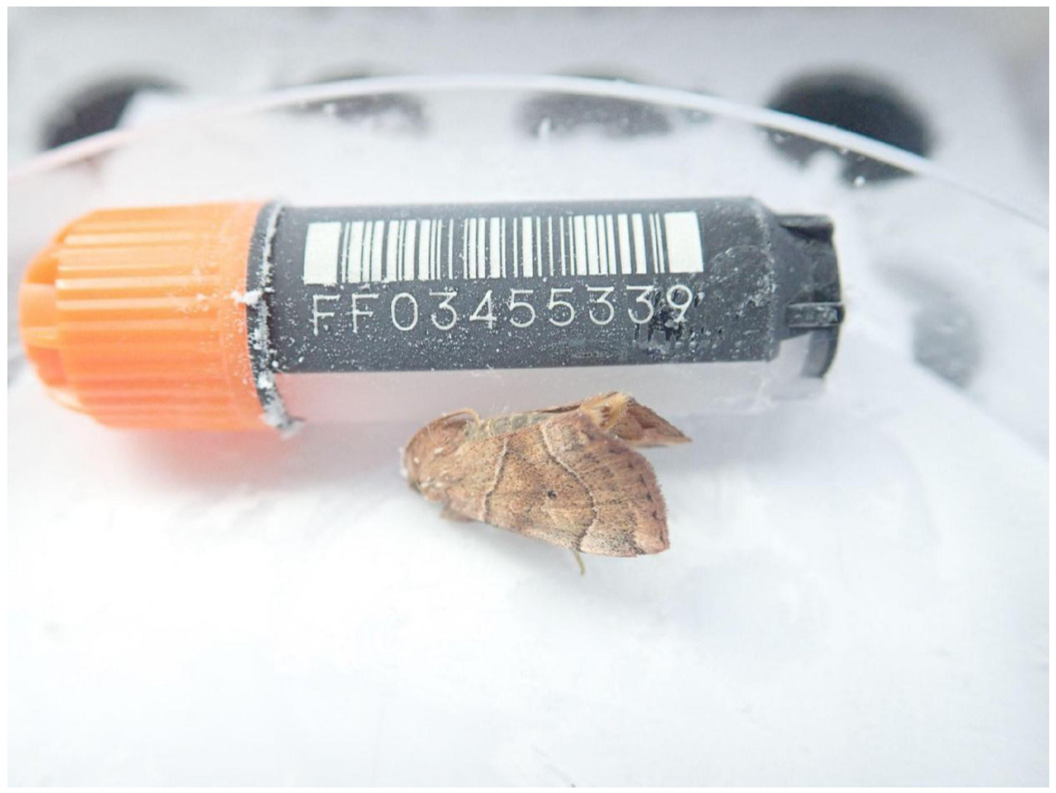
Image of the *Cosmia trapezina* specimen taken prior to preservation and processing.

The genome was sequenced from a single male *C. trapezina* collected near Ant Hills, Wytham, UK ( Figure 1). A total of 43-fold coverage in Pacific Biosciences single-molecule HiFi long reads and 42-fold coverage in 10X Genomics read clouds were generated. Primary assembly contigs were scaffolded with chromosome conformation Hi-C data. Manual assembly curation corrected 93 missing/misjoins and removed 19 haplotypic duplications, reducing the assembly size by 0.78% and the scaffold number by 51.56%, and increasing the scaffold N50 by 7.36%.

**Figure 2.**
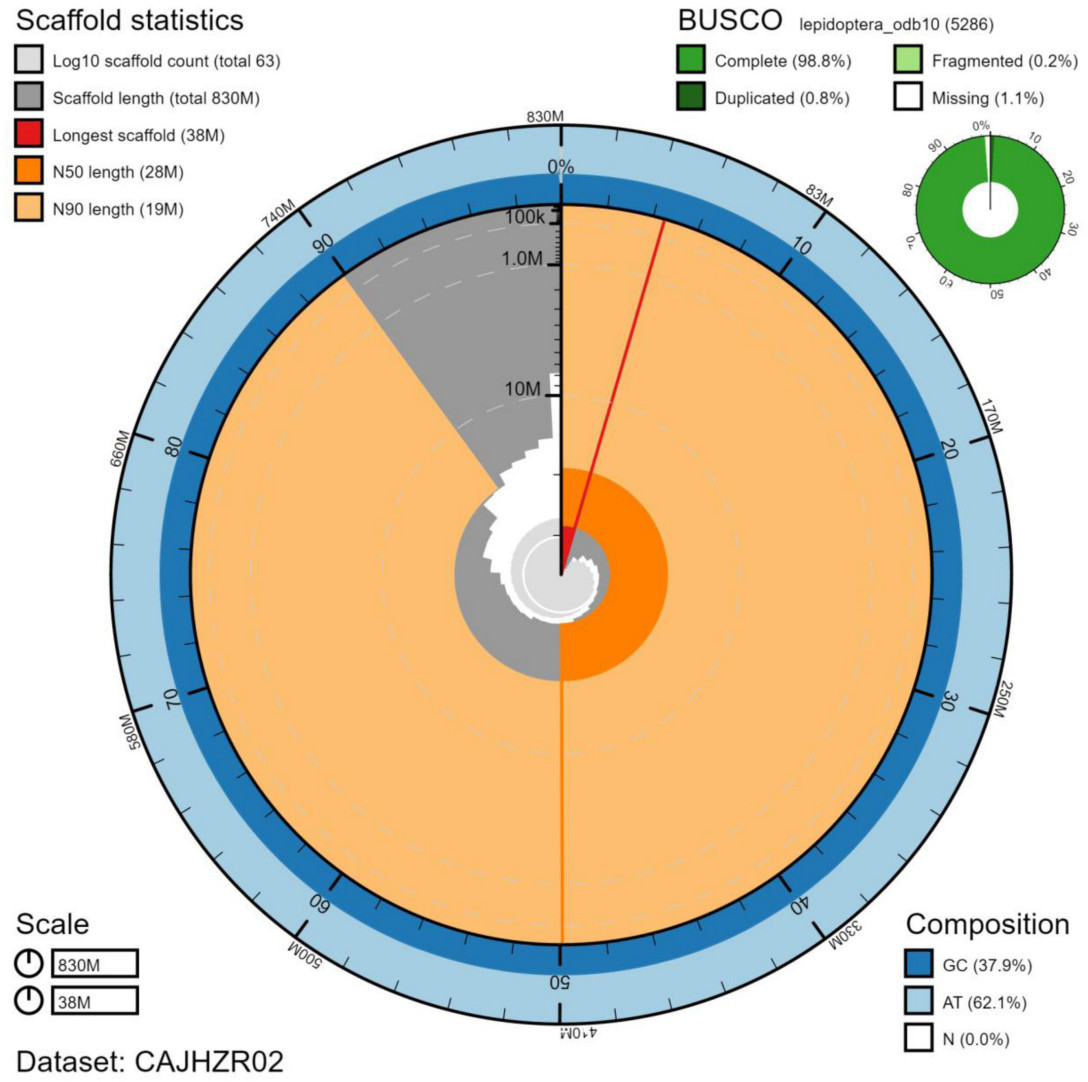
Genome assembly of *Cosmia trapezina*, ilCosTrap1.2: metrics. The BlobToolKit Snailplot shows N50 metrics and BUSCO gene completeness. The main plot is divided into 1,000 size-ordered bins around the circumference with each bin representing 0.1% of the 825,187,588 bp assembly. The distribution of chromosome lengths is shown in dark grey with the plot radius scaled to the longest chromosome present in the assembly (37,523,413 bp, shown in red). Orange and pale-orange arcs show the N50 and N90 chromosome lengths (28,022,459 and 19,015,461 bp), respectively. The pale grey spiral shows the cumulative chromosome count on a log scale with white scale lines showing successive orders of magnitude. The blue and pale-blue area around the outside of the plot shows the distribution of GC, AT and N percentages in the same bins as the inner plot. A summary of complete, fragmented, duplicated and missing BUSCO genes in the lepidoptera_odb10 set is shown in the top right. An interactive version of this figure is available at https://blobtoolkit.genomehubs.org/view/ilCosTrap1.2/dataset/CAJHZR02/snail#Filters.

The final assembly has a total length of 825 Mb in 62 sequence scaffolds with a scaffold N50 of 28.02 Mb ( Table 1). The majority, 99.87%, of the assembly sequence was assigned to 32 chromosomal-level scaffolds, representing 31 autosomes (numbered by sequence length) and the Z sex chromosome ( Figure 2– Figure 5; Table 2).

**Table 1.** Genome data for *Cosmia trapezina*, ilCosTrap1.2.

*Project accession data*	
Assembly identifier	ilCosTrap1.2
Species	*Cosmia trapezina*
Specimen	ilCosTrap1 (genome assembly; Hi-C)
NCBI taxonomy ID	116126
BioProject	PRJEB42119
BioSample ID	SAMEA7519851
Isolate information	Male, abdomen/thorax tissue (genome assembly); head tissue (Hi-C)
*Raw data accessions*	
PacificBiosciences SEQUEL II	ERR6558181-ERR6558183
10X Genomics Illumina	ERR6002608-ERR6002611
Hi-C Illumina	ERR6002612-ERR6002614
*Genome assembly*	
Assembly accession	GCA_905163495.2
*Accession of alternate haplotype*	GCA_905163595.1
Span (Mb)	825
Number of contigs	266
Contig N50 length (Mb)	7.6
Number of scaffolds	62
Scaffold N50 length (Mb)	28.02
Longest scaffold (Mb)	35.87
BUSCO * genome score	C:98.8%[S:97.9%,D:0.8%],F:0.2%, M:1.1%,n:5286

*BUSCO scores based on the lepidoptera_odb10 BUSCO set using v5.2.2. C= complete [S= single copy, D=duplicated], F=fragmented, M=missing, n=number of orthologues in comparison. A full set of BUSCO scores is available at https://blobtoolkit.genomehubs.org/view/ilCosTrap1.2/dataset/CAJHZR02/busco#Filters.

**Figure 3.**
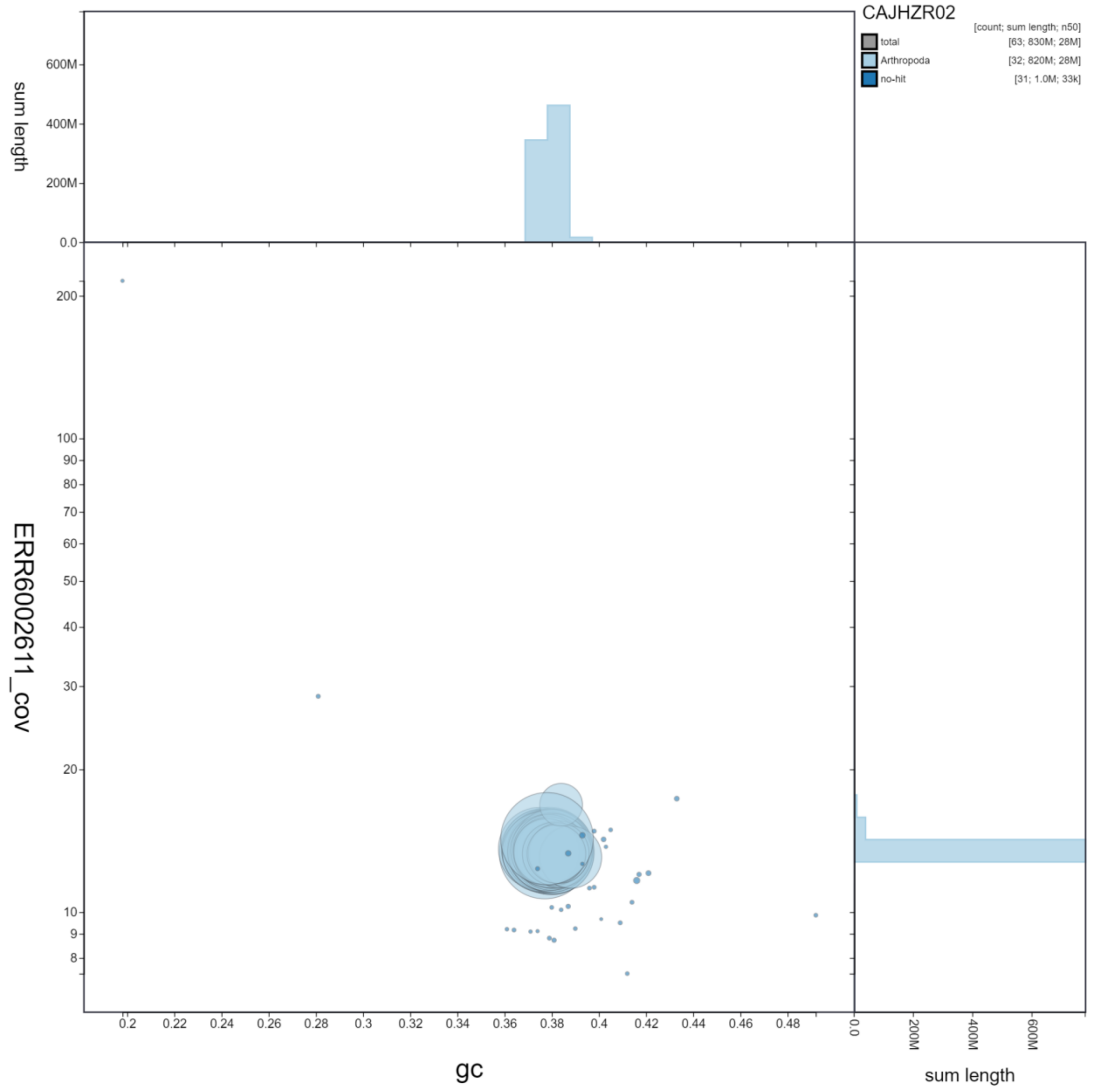
Genome assembly of *Cosmia trapezina*, ilCosTrap1.2: GC coverage. BlobToolKit GC-coverage plot. Scaffolds are coloured by phylum. Circles are sized in proportion to scaffold length. Histograms show the distribution of scaffold length sum along each axis. An interactive version of this figure is available at https://blobtoolkit.genomehubs.org/view/ilCosTrap1.2/dataset/CAJHZR02/blob#Filters.

**Figure 4.**
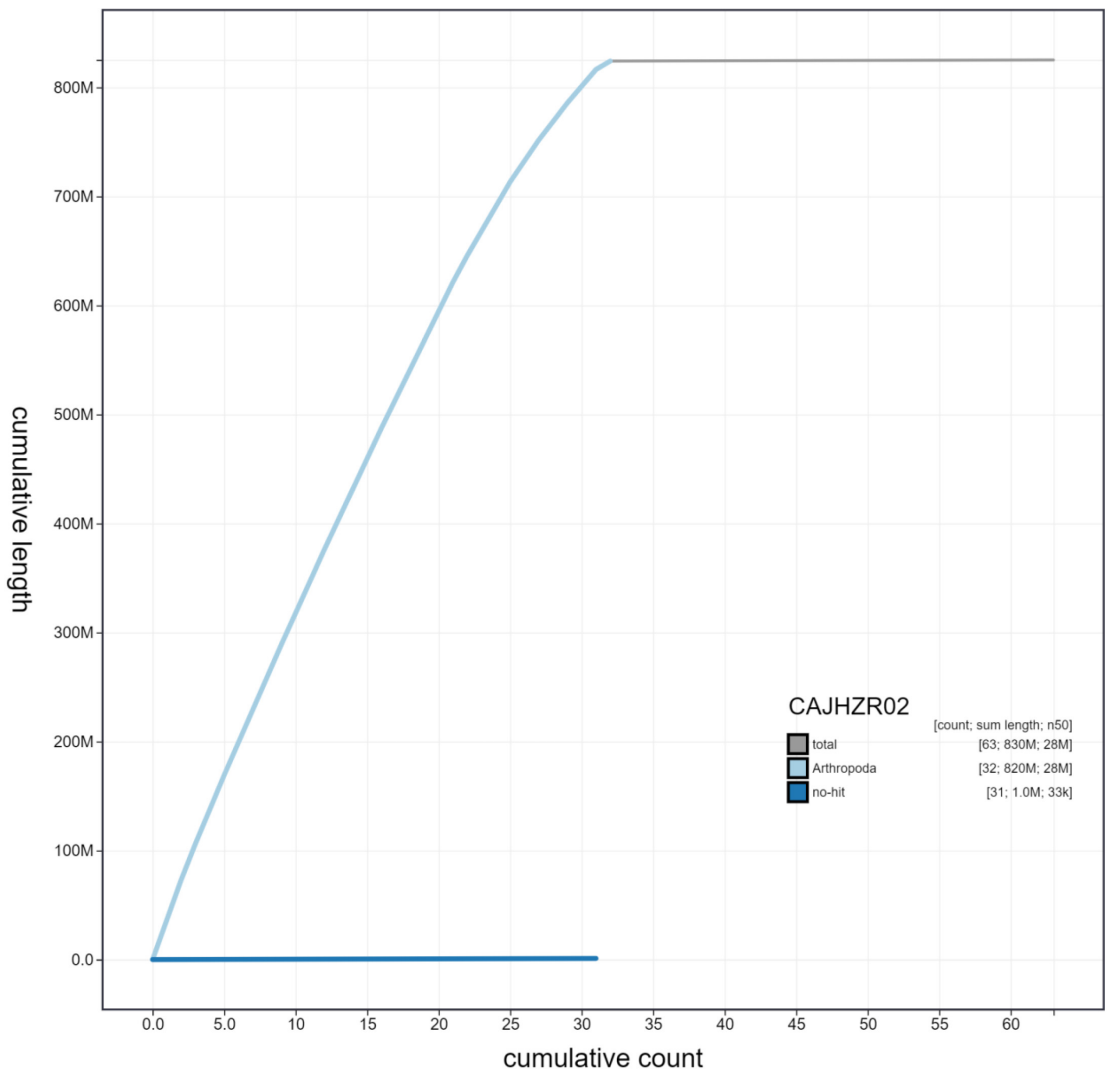
Genome assembly of *Cosmia trapezina*, ilCosTrap1.2: cumulative sequence. BlobToolKit cumulative sequence plot. The grey line shows cumulative length for all scaffolds. Coloured lines show cumulative lengths of scaffolds assigned to each phylum using the buscogenes taxrule. An interactive version of this figure is available at https://blobtoolkit.genomehubs.org/view/ilCosTrap1.2/dataset/CAJHZR02/cumulative#Filters.

**Figure 5.**
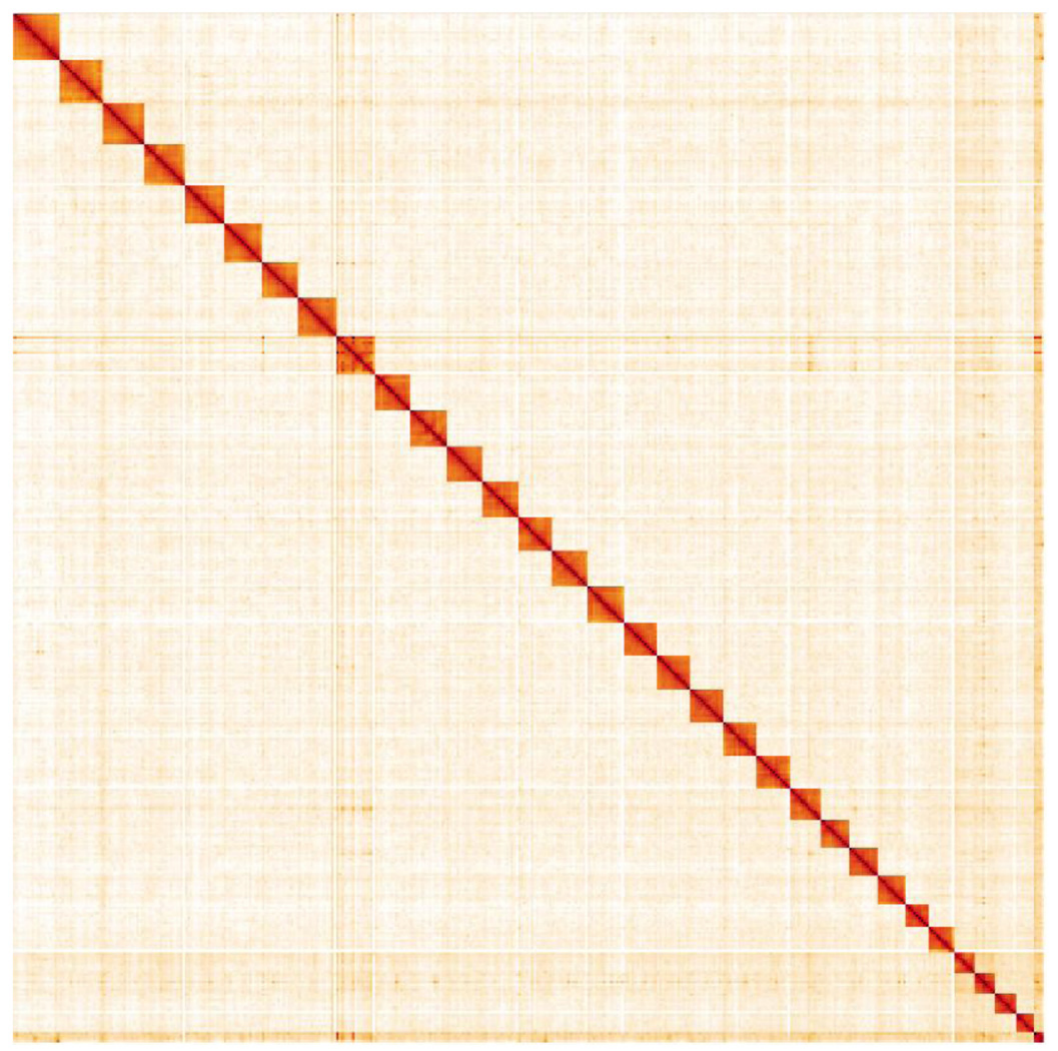
Genome assembly of *Cosmia trapezina*, ilCosTrap1.2: Hi-C contact map. Hi-C contact map of the ilCosTrap1.2 assembly, visualised in HiGlass. Chromosomes are arranged in size order from left to right and top to bottom. The interactive Hi-C map can be viewed at https://genome-note-higlass.tol.sanger.ac.uk/l/?d=DMmRE_dnRj2x5LElHv538A.

**Table 2.** Chromosomal pseudomolecules in the genome assembly of *Cosmia trapezina*, ilCosTrap1.2.

INSDC accession	Chromosome	Size (Mb)	GC%
LR991020.1	1	35.87	37.7
LR991021.1	2	33.09	37.9
LR991022.1	3	31.67	38
LR991023.1	4	30.74	38
LR991024.1	5	30.25	37.7
LR991025.1	6	30.06	37.5
LR991026.1	7	29.98	37.8
LR991027.1	8	29.62	37.9
LR991028.1	9	29.4	38
LR991029.1	10	28.86	37.6
LR991030.1	11	28.65	37.9
LR991031.1	12	28.12	37.7
LR991032.1	13	28.02	37.8
LR991033.1	14	27.94	37.6
LR991034.1	15	27.85	37.9
LR991035.1	16	27.24	38.1
LR991036.1	17	27	38
LR991037.1	18	26.84	37.8
LR991038.1	19	26.61	38
LR991039.1	20	26.03	38
LR991040.1	21	24.38	38.1
LR991041.1	22	22.86	37.8
LR991042.1	23	22.81	37.9
LR991043.1	24	22.08	37.9
LR991044.1	25	19.21	38.3
LR991045.1	26	19.02	37.8
LR991046.1	27	17.32	38.1
LR991047.1	28	16.75	38.8
LR991048.1	29	15.86	38.3
LR991049.1	30	14.8	38.2
LR991050.1	31	7.69	38.4
LR991019.1	Z	37.52	37.8
LR991051.2	MT	0.02	19.9
-	Unplaced	1.03	39.7

The assembly has a BUSCO v5.2.2 ( Manni *et al.*, 2021) completeness of 98.8% (single 97.9%, duplicated 0.8%) using the lepidoptera_odb10 reference set (n=954). While not fully phased, the assembly deposited is of one haplotype. Contigs corresponding to the second haplotype have also been deposited.

## Methods

### Sample acquisition and nucleic acid extraction

A single male *C. trapezina* specimen (ilCosTrap1) was collected using a light trap near Ant Hills, Wytham, UK (latitude 51.764, longitude -1.327) by Douglas Boyes (University of Oxford). The specimen was identified by Douglas Boyes and snap-frozen on dry ice.

DNA was extracted at the Tree of Life laboratory, Wellcome Sanger Institute. The ilCosTrap1 sample was weighed and dissected on dry ice with tissue set aside for Hi-C sequencing. Thorax and abdomen tissue was disrupted using a Nippi Powermasher fitted with a BioMasher pestle. Fragment size analysis of 0.01–0.5 ng of DNA was then performed using an Agilent FemtoPulse. High molecular weight (HMW) DNA was extracted using the Qiagen MagAttract HMW DNA extraction kit. Low molecular weight DNA was removed from a 200-ng aliquot of extracted DNA using 0.8X AMpure XP purification kit prior to 10X Chromium sequencing; a minimum of 50 ng DNA was submitted for 10X sequencing. HMW DNA was sheared into an average fragment size between 12–20 kb in a Megaruptor 3 system with speed setting 30. Sheared DNA was purified by solid-phase reversible immobilisation using AMPure PB beads with a 1.8X ratio of beads to sample to remove the shorter fragments and concentrate the DNA sample. The concentration of the sheared and purified DNA was assessed using a Nanodrop spectrophotometer and Qubit Fluorometer and Qubit dsDNA High Sensitivity Assay kit. Fragment size distribution was evaluated by running the sample on the FemtoPulse system.

### Sequencing

Pacific Biosciences HiFi circular consensus and 10X Genomics Chromium read cloud sequencing libraries were constructed according to the manufacturers’ instructions. Sequencing was performed by the Scientific Operations core at the Wellcome Sanger Institute on Pacific Biosciences SEQUEL II (HiFi) and Illumina HiSeq (10X) instruments. Hi-C data were generated in the Tree of Life laboratory from remaining head tissue of ilCosTrap1 using the Arima v1 kit and sequenced on a Illumina HiSeq (10X) instrument.

### Genome assembly

Assembly was carried out with HiCanu ( Nurk *et al.*, 2020); haplotypic duplication was identified and removed with purge_dups ( Guan *et al.*, 2020). One round of polishing was performed by aligning 10X Genomics read data to the assembly with longranger align, calling variants with freebayes ( Garrison & Marth, 2012). The assembly was then scaffolded with Hi-C data ( Rao *et al.*, 2014) using SALSA2 ( Ghurye *et al.*, 2019). The assembly was checked for contamination and corrected using the gEVAL system ( Chow *et al.*, 2016) as described previously ( Howe *et al.*, 2021). Manual curation ( Howe *et al.*, 2021) was performed using gEVAL, HiGlass ( Kerpedjiev *et al.*, 2018) and Pretext. The mitochondrial genome was assembled using MitoHiFi ( Uliano-Silva *et al.*, 2021), which performs annotation using MitoFinder ( Allio *et al.*, 2020). The genome was analysed and BUSCO scores generated within the BlobToolKit environment ( Challis *et al.*, 2020). Table 3 contains a list of all software tool versions used, where appropriate.

**Table 3.** Software tools used.

Software tool	Version	Source
HiCanu	2.1	Nurk *et al.*, 2020
purge_dups	1.2.3	Guan *et al.*, 2020
SALSA2	2.2	Ghurye *et al.*, 2019
longranger align	2.2.2	https://support.10xgenomics.com/ genome-exome/software/pipelines/ latest/advanced/other-pipelines
freebayes	1.3.1-17- gaa2ace8	Garrison & Marth, 2012
MitoHiFi	1.0	Uliano-Silva *et al.*, 2021
HiGlass	1.11.6	Kerpedjiev *et al.*, 2018
PretextView	0.2.x	https://github.com/wtsi-hpag/PretextView
BlobToolKit	3.0.5	Challis *et al.*, 2020

### Ethics/compliance issues

The materials that have contributed to this genome note have been supplied by a Darwin Tree of Life Partner. The submission of materials by a Darwin Tree of Life Partner is subject to the Darwin Tree of Life Project Sampling Code of Practice. By agreeing with and signing up to the Sampling Code of Practice, the Darwin Tree of Life Partner agrees they will meet the legal and ethical requirements and standards set out within this document in respect of all samples acquired for, and supplied to, the Darwin Tree of Life Project. Each transfer of samples is further undertaken according to a Research Collaboration Agreement or Material Transfer Agreement entered into by the Darwin Tree of Life Partner, Genome Research Limited (operating as the Wellcome Sanger Institute), and in some circumstances other Darwin Tree of Life collaborators.

## Data availability

European Nucleotide Archive: Cosmia trapezina (dun-bar pinion). Accession number PRJEB42119; https://identifiers.org/ena.embl/PRJEB42119.

The genome sequence is released openly for reuse. The *C. trapezina* genome sequencing initiative is part of the Darwin Tree of Life (DToL) project. All raw sequence data and the assembly have been deposited in INSDC databases. The genome will be annotated and presented through the Ensembl pipeline at the European Bioinformatics Institute. Raw data and assembly accession identifiers are reported in Table 1.

## Author information

Members of the University of Oxford and Wytham Woods Genome Acquisition Lab are listed here: https://doi.org/10.5281/zenodo.6418202.

Members of the Darwin Tree of Life Barcoding collective are listed here: https://doi.org/10.5281/zenodo.6418156.

Members of the Wellcome Sanger Institute Tree of Life programme are listed here: https://doi.org/10.5281/zenodo.6418327.

Members of Wellcome Sanger Institute Scientific Operations: DNA Pipelines collective are listed here: https://doi.org/10.5281/zenodo.5746904.

Members of the Tree of Life Core Informatics collective are listed here: https://doi.org/10.5281/zenodo.6125046.

Members of the Darwin Tree of Life Consortium are listed here: https://doi.org/10.5281/zenodo.6418363.
