# Supplementary material for: The genome sequence of the dun-bar pinion, Cosmia trapezina (Linnaeus, 1758)
Source: Wellcome Open Res. 2022 Jul 13;7:189. [Version 1] doi: 10.12688/wellcomeopenres.17925.1 (PMC10517310; doi:10.12688/wellcomeopenres.17925.1)
Supplement: Peer review history

# Reviewer report: Reviewer response for version 1

**DOI:** 10.21956/wellcomeopenres.19863.r67218

**Published:** 2023-09-22

**Authors:** Camille Meslin

**Affiliations:** 1 INRAE, CNRS, IRD, UPEC, University of Paris, Paris, France

This is a well-written data note on the genome of the dun-bar pinion Cosmia trapezina and a great resource given the high quality of the genome.

 My only interrogation about this genome is whether some kind of annotation was performed on it? It would be a great addition for people interested in comparative genomics.

Are sufficient details of methods and materials provided to allow replication by others?

Yes

Is the rationale for creating the dataset(s) clearly described?

Yes

Are the datasets clearly presented in a useable and accessible format?

Yes

Are the protocols appropriate and is the work technically sound?

Yes

Reviewer Expertise:

Chemosensory receptors, comparative genomics, evolution

I confirm that I have read this submission and believe that I have an appropriate level of expertise to confirm that it is of an acceptable scientific standard.

# Reviewer report: Reviewer response for version 1

**DOI:** 10.21956/wellcomeopenres.19863.r52447

**Published:** 2022-10-06

**Authors:** Amali Thrimawithana

**Affiliations:** 1 Plant and Food Research Institute of New Zealand Ltd, Auckland, New Zealand

Well written data note on the dun-bar genome. Protocols for generating the assembly seem appropriate and have constructed high quality genome for the species. All versions and assembly methods are followable - one query: was it wasn't clear which tool was used for one round of polishing (though I understand longranger was used for aligning). 

 Another was, was the 10x data only used for correction - were no further scaffolding/assembly with it trialled? 

 I was also curious to know if the heterozygosity level etc was examined with kmer analysis of sort?

 Again, great resource to have for the species.

Are sufficient details of methods and materials provided to allow replication by others?

Yes

Is the rationale for creating the dataset(s) clearly described?

Yes

Are the datasets clearly presented in a useable and accessible format?

Yes

Are the protocols appropriate and is the work technically sound?

Yes

Reviewer Expertise:

Genomics, transcriptomics, metagenomics in plant insect and fish

I confirm that I have read this submission and believe that I have an appropriate level of expertise to confirm that it is of an acceptable scientific standard.
